# Effects of Dantrolene on Arrhythmogenicity in Isolated Regional Ischemia-Reperfusion Rabbit Hearts with or without Pacing-Induced Heart Failure

**DOI:** 10.1155/2015/532820

**Published:** 2015-02-19

**Authors:** Chung-Chuan Chou, Hui-Ling Lee, Po-Cheng Chang, Hung-Ta Wo, Ming-Shien Wen, San-Jou Yeh

**Affiliations:** ^1^Division of Cardiology, Department of Medicine, Chang Gung Memorial Hospital, Linkou District, Taiwan; ^2^Chang Gung University College of Medicine, Taoyuan City, Taiwan; ^3^Department of Anesthesia, Chang Gung Memorial Hospital, Taipei, Taiwan

## Abstract

Dantrolene was reported to suppress ventricular fibrillation (VF) in failing hearts with acute myocardial infarction, but its antiarrhythmic efficacy in regional ischemia-reperfusion (IR) hearts remains debatable. Heart failure (HF) was induced by right ventricular pacing. The IR rabbit model was created by coronary artery ligation for 30 min, followed by reperfusion for 15 min in vivo in both HF and non-HF groups (*n* = 9 in each group). Simultaneous voltage and intracellular Ca^2+^ (Ca_i_) optical mapping was then performed in isolated Langendorff-perfused hearts. Electrophysiological studies were conducted and VF inducibility was evaluated by dynamic pacing. Dantrolene (10 *μ*M) was administered after baseline studies. The HF group had a higher VF inducibility than the control group. Dantrolene had both antiarrhythmic (prolonged action potential duration (APD) and effective refractory period) and proarrhythmic effects (slowed conduction velocity, steepened APD restitution slope, and enhanced arrhythmogenic alternans induction) but had no significant effects on ventricular premature beat (VPB) suppression and VF inducibility in both groups. A higher VF conversion rate in the non-HF group was likely due to greater APD prolonging effects in smaller hearts compared to the HF group. The lack of significant effects on VPB suppression by dantrolene suggests that triggered activity might not be the dominant mechanism responsible for VPB induction in the IR model.

## 1. Introduction

Reperfusion therapy by thrombolysis or immediate percutaneous coronary intervention is commonly performed for acute myocardial infarction (AMI). However, fatal arrhythmia may occur not only during ischemia but also during reperfusion. It has been reported that intracellular Ca^2+^ (Ca_i_) overload is involved in the development of fatal arrhythmias postischemia-reperfusion (IR) injury [1, 2]. The elevated Ca_i_ in IR myocardium may enhance triggered activity [3], thereby contributing to IR arrhythmias [4]. Both IR and heart failure (HF) impair sarcoplasmic reticulum (SR) Ca^2+^ ATPase pump activity [5, 6], which results in decreased SR Ca^2+^ load and elevated diastolic Ca^2+^ to facilitate afterdepolarizations induction and triggered activity [7]. It is reasonable to speculate that prevention of spontaneous SR Ca^2+^ leak may improve Ca_i_ homeostasis and reduce IR arrhythmias, especially in HF. Dantrolene, a stabilizer of skeletal muscle ryanodine receptor-1, has been reported to suppress diastolic Ca^2+^ leakage by stabilizing ryanodine receptor-2 of failing cardiomyocytes [8]. We previously reported that dantrolene is effective in preventing ventricular fibrillation (VF) storm by suppressing ventricular premature beats (VPBs) in isolated failing rabbit hearts with AMI [9]. Ortiz et al. reported that dantrolene protects the myocardium against myocardial stunning and IR arrhythmias in Wistar rat hearts [10]; however, other studies have reported that dantrolene has no significant effects on IR arrhythmia in canine hearts [11] and on IR injury in rabbits in vivo [12]. Thus, the effect of dantrolene on arrhythmogenesis in IR hearts remains debatable. The purpose of this study was to examine whether dantrolene has a primary antiarrhythmic effect and the mode of its action in isolated IR rabbit hearts with or without pacing-induced HF. We performed simultaneous membrane voltage (*V*_*m*_) and Ca_i_ mapping to investigate the electrophysiological and Ca_i_ changes during dantrolene infusion in Langendorff-perfused rabbit hearts with IR injury.

## 2. Materials and Methods

The research protocol was approved by the Institutional Animal Care and Use Committee of Chang Gung Memorial Hospital (approval number 2009092201) and conformed to the Guide for the Care and Use of Laboratory Animals published by the United States National Institutes of Health. Twenty-two adult New Zealand white rabbits (2.8–4.2 kg) were used in the study. Two rabbits died within 4 weeks of pacing initiation, and the hearts from the remaining 20 rabbits (11 with pacing-induced HF (the HF group) and 9 without pacemaker implantation (non-HF group)) were harvested for IR induction. Two rabbits in the HF group developed VF storm during the ischemia period and were not used for optical mapping studies. Left ventricular (LV) function was assessed by echocardiography at baseline and after 4 weeks of pacing.

### 2.1. Pacing-Induced HF and IR Model Creation

Rapid right ventricular pacing was used to induce HF as described previously [9]. The IR model was created by the same method used in our previous study [13]. Briefly, the rabbits were premedicated with intramuscular injection of ketamine (35 mg/kg) and xylazine (5 mg/kg), intubated and anesthetized with isoflurane. When the rabbits were fully anesthetized and unresponsive to physical stimuli, the chests were opened via a left thoracotomy. An obtuse marginal branch of the left circumflex artery was then ligated halfway between the atrioventricular groove and the cardiac apex for 30 minutes, followed by reperfusion. The limb lead electrocardiogram (ECG) was monitored continuously.

### 2.2. Optical Mapping of Isolated Rabbit Hearts

After reperfusion for 15 minutes, the hearts were excised and Langendorff-perfused with 37°C Tyrode's solution (in mmol/L: NaCl 125, KCl 4.5, MgCl_2_ 0.25, NaHCO_3 _24, NaH_2_PO_4 _1.8, CaCl_2 _1.8, glucose 5.5, and albumin 50 mg/L) and equilibrated with 95% O_2_ and 5% CO_2_ to maintain a pH of 7.4. The coronary perfusion pressure was regulated and maintained at 70–80 cmH_2_O. The hearts were stained with Rhod-2_AM_ (Molecular Probes, Eugene, OR, USA) for Ca_i_ and RH237 (Molecular Probes) for *V*_*m*_ mapping. The double-stained hearts were illuminated with a laser at 532 nm wavelength. The emitted fluorescence was filtered and acquired simultaneously with two charge-coupled device cameras (CA-D1-0128T; Dalsa Inc., Billerica, MA, USA) at 269 frames/second. Digital images (128 × 128 pixels) were gathered from the epicardium of the LV (25 × 25 mm^2^ area), resulting in a spatial resolution of 0.2 × 0.2 mm^2^ per pixel. Motion artifacts were suppressed by 5 *μ*M cytochalasin D. The average fluorescence level ($\bar{F}$) of an individual pixel was first calculated for the duration of recording. The ratio on each pixel was then calculated as $(F-\bar{F})/\bar{F}$, color-coded with shades of red (depolarization) or blue (repolarization), and animated to show propagation patterns in the mapping field.

### 2.3. Experimental Protocols

A bipolar catheter was inserted into the right ventricular apex for pacing at twice the threshold. The effective refractory period (ERP) was measured by giving a premature stimulus after 8 beats at a 400 ms pacing cycle length (PCL). APD_80_ (APD at 80% repolarization) restitution curve was constructed, and APD_80_ and Ca_i_ alternans were induced by a dynamic pacing protocol [14]. VF inducibility was defined as the ability to provoke sustained VF (>2 min) with the dynamic pacing protocol [15]. If VF was not induced by dynamic pacing, burst pacing (PCL of 70–90 ms, 5 ms pulse width, and 5 mA current for 3–8 seconds) was used. Three episodes of VF (4 seconds each) were obtained consecutively for dominant frequency (DF) analysis. Defibrillation using epicardial patch electrodes was performed for sustained VF. After the baseline study, dantrolene (10 *μ*M) was administered for 20 minutes, and the experimental protocol was repeated.

### 2.4. Data Analysis

APD_80_ restitution curves were constructed at selected evenly spaced sites [14]. The maximum slope of APD_80_ restitution curve was determined after curve fitting by first-order exponential fitting. We used monoexponential fitting to compute the time constant (*τ*) of the decay portion of the Ca^2+^ transient [16] at a PCL of 400 ms. The thresholds of APD_80_ and Ca_i_ alternans were defined as the longest PCL required to produce a 10 ms difference in APD_80_ and a 10% difference in Ca_i_ amplitude between consecutive beats, respectively [17]. The phase was considered positive for a short-long APD and a small-large Ca_i_ amplitude sequence (color-coded in red) and was negative for a long-short APD and a large-small Ca_i_ amplitude sequence (color-coded in green). Spatially discordant alternans (SDA) was evidenced by the presence of both red and green regions separated by a nodal line. The SDA threshold was defined as the longest PCL required to reach the alternans threshold on both sides of a nodal line. To estimate conduction velocity (CV), we measured the distance and conduction time between the earliest activation point and two epicardial points in the non-IR and IR zones, respectively [13]. VPB burden (beats/5 min) was calculated by pseudo-ECG recordings at 0–5 min (taken as baseline), 5–10 min, 10–15 min, and 15–20 min during dantrolene infusion to evaluate its effects on VPB suppression.

### 2.5. Statistics

Continuous variables were expressed as mean ± standard deviation. One-way repeated measures ANOVA (with post hoc LSD analysis) was performed to evaluate the effects of dantrolene on VPB suppression during the 20-minute drug infusion period. Two-way repeated measures ANOVA was performed to evaluate the effects of dantrolene on APD_80_ (PCL at 300, 200, and 150 ms) and CV (PCL at 300, 200, 150, and 120 ms). The McNemar test was used to compare the VF inducibility at baseline and after dantrolene infusion. A paired* t*-test was used to evaluate statistical significance in ERP, the mean maximum slope of APD restitution, Ca_i_ decay, the threshold of alternans and SDA, and the mean maximum DF (DF_max⁡_) of VF before and after dantrolene treatment in the same hearts. An unpaired* t*-test was performed to compare the CV, APD_80_, and Ca_i_ decay between the HF and non-HF groups and between the non-IR and IR zones. Generalized estimating equation was used to examine whether the effect of dantrolene on APD_80_ was significantly different between the HF and non-HF groups. Differences were considered significant when *P* < 0.05.

## 3. Results

In the HF group, the mean LV ejection fraction was decreased from 62 ± 6% to 29 ± 7% after 4-week rapid pacing (*P* < 0.01, *n* = 9). In the non-HF group, the mean LV ejection fraction was 66 ± 6% (*n* = 9). The wet weight of the hearts in the HF group (21 ± 4 g, *n* = 9) was significantly heavier than the non-HF group (12 ± 2 g, *n* = 9, *P* < 0.001).

### 3.1. Effects of Dantrolene on VPB

**Figure 1:**
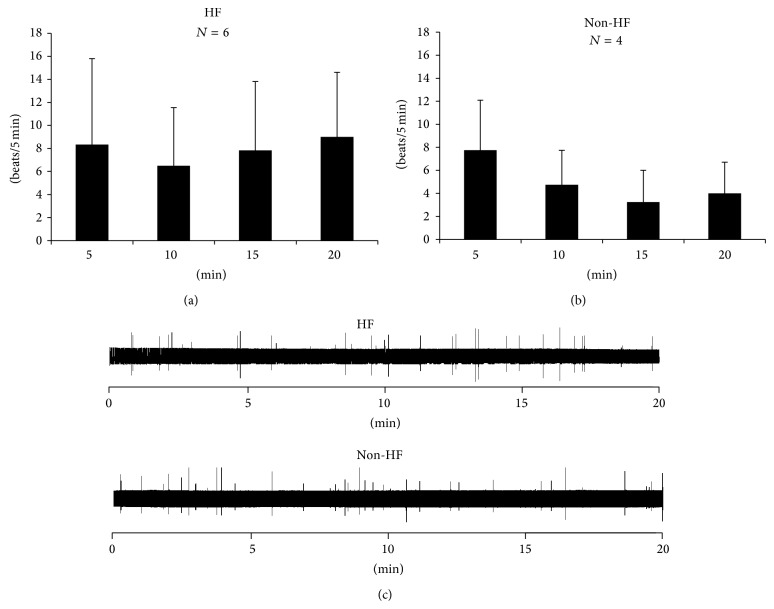
Dantrolene had no significant effects on suppressing VPBs in the HF (a) and non-HF (b) groups. (c) pseudo-ECG showed no significant decrease of VPBs density (spikes) during dantrolene infusion in either group.

In the HF group, 6 of the 9 rabbit hearts had frequent VPBs at baseline (8.3 ± 7.5 beats/5 min); in the non-HF group, 4 of the 9 rabbits hearts had frequent VPBs at baseline (7.8 ± 4.3 beats/5 min). There was no significant difference of baseline VPB burden between two groups (*P* > 0.05). Dantrolene did not lower the frequency of VPB in either groups (Figures 1(a) and 1(b), *P* > 0.05 for all comparisons).  Figure 1(c) shows examples of pseudo-ECG tracings of a failing (upper) and a control (bottom) heart. The VPB burdens were 9, 8, 10, and 9 beats/5 min (HF) and 12, 8, 6, and 8 beats/5 min (non-HF) at 0–5, 5–10, 10–15, and 15–20 min, respectively.

### 3.2. Electrophysiological Responses to Dantrolene Administration

#### 3.2.1. APD_80_ and ERP

The HF group had a significantly longer APD_80_ than the control group at PCL = 300 ms (*P* = 0.03) (Table 1). In the HF group, dantrolene prolonged APD_80_ at PCL = 300 ms (*P* = 0.03), 200 ms (*P* = 0.03), and 150 ms (*P* = 0.02) and the ERP (*P* = 0.03). In the non-HF group, dantrolene prolonged APD_80_ at PCL = 300 ms (*P* = 0.003), 200 ms (*P* = 0.001), and 150 ms (*P* = 0.004) and the ERP (*P* = 0.002) (Table 1). Generalized estimating equation analysis showed that there was a trend that dantrolene exerted a greater effect on APD_80_ prolongation in the non-HF group than in the HF group at PCL = 150 ms (*P* = 0.06). There was no significant difference in APD_80 _between the non-IR and IR zones at baseline or with dantrolene at all PCLs in either group (*P* > 0.05 for all comparisons) (Table 2).

#### 3.2.2. APD_80_ Restitution Slope

**Figure 2:**
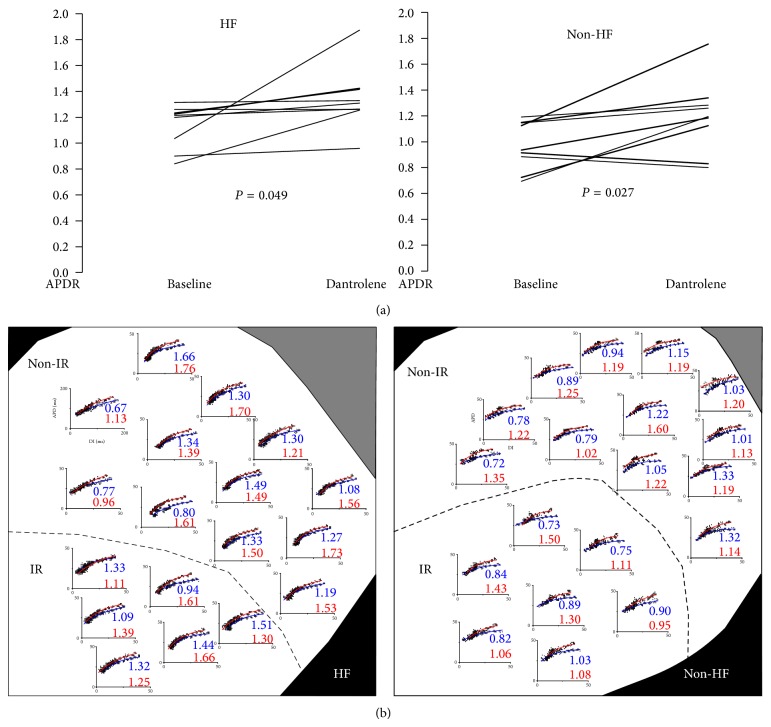
Dantrolene steepened APD_80_ restitution slopes in both groups. (a) Paired statistics for APD restitution (APDR) slopes. The data points (lines) are averages from each experiment. (b) representative examples of the spatial distribution of APDR curves at baseline (blue) and after dantrolene infusion (red) in both groups. The numbers under each subpanel are the maximum slopes of APD_80_ restitution at the selected evenly-spaced sites. DI: diastolic interval.

There was a tendency of a steeper mean maximum slope of APD_80_ restitution in the HF group than in the non-HF group (*P* = 0.07, Table 1). There was no significant difference in the mean APD restitution slopes between IR and non-IR at baseline and after dantrolene infusion in either groups (*P* > 0.05 for all comparisons, Table 2). Dantrolene steepened the maximum slope of APD_80_ restitution in the HF group (*P* = 0.049) and the non-HF group (*P* = 0.027) (Figure 2(a)).  Figure 2(b) shows an example. In a failing heart (left subpanel), the mean maximal APD_80_ restitution slopes were 1.21 ± 0.27 at baseline (blue) versus 1.44 ± 0.23 after dantrolene infusion (red) (*P* = 0.004, *n* = 18 points); in a non-HF heart (right subpanel), dantrolene also significantly steepened APD_80_ restitution slope (0.96 ± 0.19 versus 1.21 ± 0.16, *P* < 0.001, *n* = 19 points).

#### 3.2.3. CV

**Figure 3:**
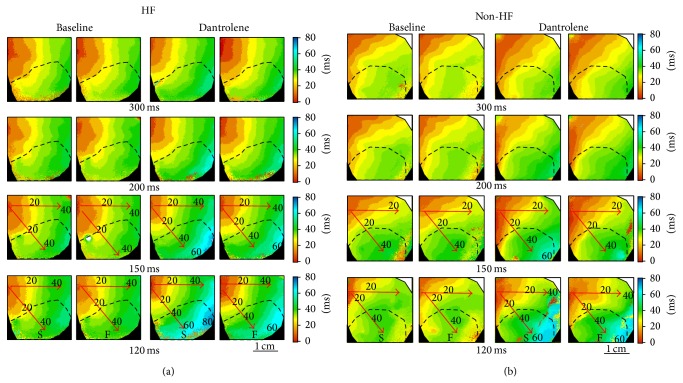
Effects of dantrolene on CV and anisotropic conduction. (a) and (b) are representative examples of isochronal maps in the HF and non-HF groups, respectively. Rate-dependent conduction slowing is shown in both groups. The number under each subpanel is the PCL. Dashed lines indicate the border of IR zone; red arrows indicate the directions of wavefront propagation. S: slow; F: fast.

The HF group had a slower CV than the non-HF group at baseline (*P* = 0.01, 0.02, 0.02, <0.001 at PCLs = 300, 200, 150, and 120 ms, resp.). Dantrolene decreased CV at all PCLs in both groups (Table 1), and the differences of CV between the HF and non-HF groups became less significant after dantrolene infusion (*P* = 0.15, 0.22, 0.30, 0.02 at PCLs = 300, 200, 150, and 120 ms, resp.). In the HF group, there was no significant difference in CV between the IR and non-IR zones at baseline, but CV became significantly slower in the IR zone than the non-IR zone as the PCL decreased to 120 ms (*P* = 0.04) after dantrolene infusion (Table 2). In the non-HF group, there was a tendency of slower CV in the IR than in the non-IR zones at baseline (*P* = 0.08, 0.1, 0.06, 0.06 at PCLs of 300, 200, 150, and 120 ms, resp.), and the differences became significant after dantrolene infusion at all PCLs (*P* = 0.03, 0.04, 0.005, 0.009 at PCLs of 300, 200, 150, and 120 ms, resp.) (Table 2).  Figure 3(a) shows an example in the HF group. Impulses propagated smoothly at PCLs >150 ms but took longer to activate the reperfused region at shorter PCLs (<150 ms), especially after dantrolene infusion. At baseline, CV (PCL = 300 ms versus 120 ms) was decreased from 77 cm/s to 62 cm/s in the non-IR zone and from 77 cm/s to 61 cm/s in the IR zone. After dantrolene infusion, CV (PCL = 300 ms versus 120 ms) was decreased from 72 cm/s to 50 cm/s in the non-IR zone and from 66 cm/s to 46 cm/s in the IR zone in this heart.  Figure 3(b) shows an example of the non-HF group. At baseline, CV (PCL = 300 ms versus 120 ms) was decreased from 93 cm/s to 72 cm/s in the non-IR zone and from 82 cm/s to 68 cm/s in the IR zone. After dantrolene infusion, CV (PCL = 300 ms versus 120 ms) was decreased from 86 cm/s to 52 cm/s in the non-IR zone and from 70 cm/s to 47 cm/s in the IR zone in this heart. In addition, CV alternans was induced by rapid pacing, which played a role in facilitating SDA induction.

#### 3.2.4. Alternans and SDA

APD_80_ and Ca^2+^ transient alternans were in synchrony in all mapped episodes (an example shown in Figure 5(c)). Dantrolene significantly lowered the pacing rate threshold for alternans induction in both groups (HF: 200 ± 30 ms (baseline) versus 222 ± 36 ms (dantrolene), *P* = 0.02, *n* = 9; non-HF: 187 ± 11 ms (baseline) versus 237 ± 30 ms (dantrolene), *P* < 0.001, *n* = 9). In addition, the threshold of SDA was lowered by dantrolene in both groups (HF: 120 ± 17 ms (baseline) versus 141 ± 16 ms (dantrolene), *P* < 0.001, *n* = 9; non-HF: 126 ± 11 ms (baseline) versus 147 ± 13 ms (dantrolene), *P* = 0.01, *n* = 7).

#### 3.2.5. Ca_i_ Decay

**Figure 4:**
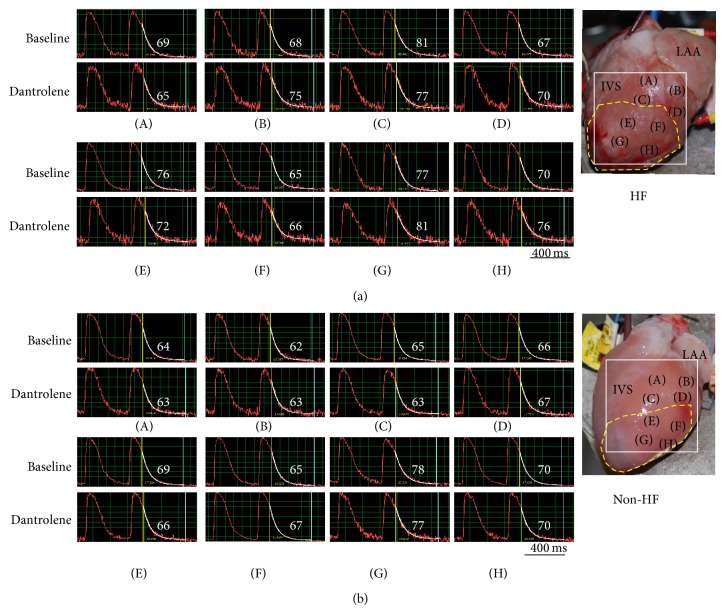
Effects of dantrolene on Ca_i_ decay. (a) and (b) representative examples of the distribution of Ca^2+^ decay time in the non-IR and IR zones at baseline and during dantrolene infusion in the HF and non-HF groups, respectively. Dantrolene had no significant effects on Ca_i_ delay in either group. The number on each subpanel is the *τ* value (ms). The right subpanel illustrates the mapping field, and (A) to (H) indicate sites where the Ca_i_ decay time was measured. LAA: left atrial appendage; IVS: interventricular sulcus.

Dantrolene had no significant effects on Ca_i_ reuptake in either group (Table 1). In the HF group, there was no significant difference in the *τ* values between the IR and non-IR zones at baseline or after dantrolene infusion (*P* > 0.05 for all comparisons) (Table 2). A representative example is provided in Figure 4(a). The mean *τ* values (non-IR versus IR) were 71 ± 7 ms and 72 ± 6 ms at baseline and 72 ± 5 ms and 74 ± 6 ms after dantrolene infusion in this failing heart. In the non-HF group, Ca_i_ decay was significantly slower in the IR zone than in the non-IR zone at baseline (*P* = 0.007) and after dantrolene infusion (*P* = 0.03) (Table 2). As shown in Figure 4(b), the mean *τ* values (non-IR versus IR) were 64 ± 2 ms and 71 ± 5 ms at baseline and 64 ± 2 ms and 70 ± 5 ms after dantrolene infusion in this non-HF heart.

### 3.3. Effects of Dantrolene on VF Induction and Maintenance

The HF group had a higher VF inducibility than the non-HF group at baseline (7 of 9 versus 2 of 9, *P* = 0.03). Almost all pacing-induced VF episodes (including burst pacing) were sustainable in the HF (9 of 9) and non-HF (8 of 9) groups at baseline. Dantrolene had no significant effects on VF inducibility (7 of 9 versus 7 of 9, *P* = NS) or VF maintenance (9 of 9 versus 6 of 9, *P* = NS) in the HF group. In the non-HF group, the VF inducibility was borderline increased (2 of 9 versus 6 of 9, *P* = 0.125), but VF became less sustainable (8 of 9 versus 3 of 9, *P* = 0.0498) after dantrolene infusion.

**Figure 5:**
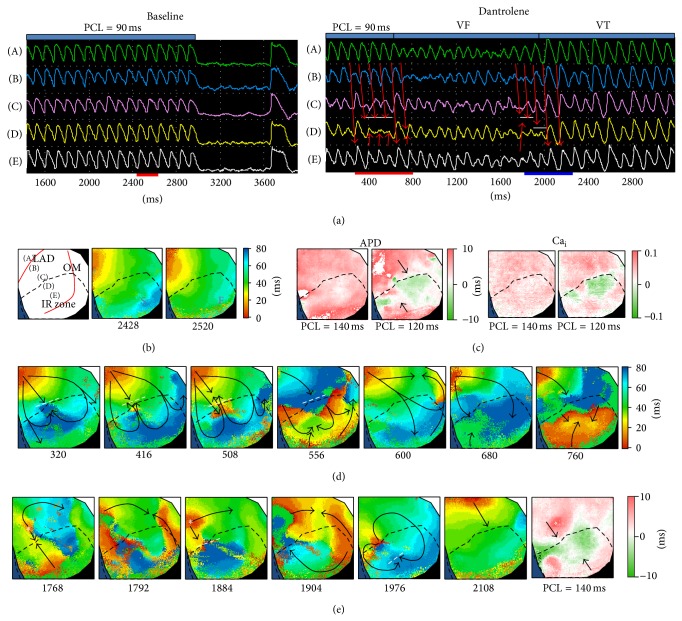
VF induction and VF conversion to VT during dantrolene administration in a failing heart. (a) *V*_*m*_ tracings corresponding to the points labeled in panel (b) during rapid pacing (PCL = 90 ms) at baseline (left subpanel) and with dantrolene (right subpanel). The red arrows indicate wavefront propagation and the white lines indicate conduction block. (b) Schematic illustration of the mapped region (left subpanel) and isochronal maps (right subpanels) corresponding to two consecutive beats labeled in panel (a) (left subpanel, red bar). (c) Ca_i_ and APD alternated in synchrony. SDA was not induced at PCL = 140 ms until it was shortened to 120 ms at baseline but was induced at PCL = 140 ms with dantrolene infusion (panel (e), right subpanel). The black arrows indicate nodal lines. (d) and (e) isochronal maps of VF initiation and VF conversion to VT corresponding to the period labeled in panel (a) (right subpanel and red and blue bars, resp.). The white dashed lines indicate functional conduction block; white asterisks indicate focal breakthrough.

Figure 5 shows an example of dantrolene effects on VF dynamics in the HF group. In this failing heart, VF was not induced during dynamic pacing at baseline (PCL = 90 ms, Figure 5(a), left subpanel), even if CV alternans (Figure 5(b)) and SDA (from PCL = 120 ms, Figure 5(c)) were induced. After dantrolene infusion, VF was induced by dynamic pacing (PCL = 90 ms) but converted to VT spontaneously (Figure 5(a), right subpanel).  Figure 5(d) shows that rapid pacing induced functional conduction block to form figure-8 reentry (frames 320, 416, and 508 ms), and the conduction delay caused wavefronts to arise from the opposite side of the IR border to activate the IR zone and collide with the original wavefronts to perpetuate VF (frame 760 ms) (see online video 1 in Supplementary Material available online at http://dx.doi.org/10.1155/2015/532820). However the VF was not sustainable. As shown in Figure 5(e), the breakthrough wavefronts could not propagate into the IR zone directly (frames 1768, 1884 ms). The functional conduction block simplified the wavefront propagation pattern and converted VF to VT (online video 2). SDA was induced at a longer PCL after dantrolene infusion (from 140 ms, Figure 5(e), right subpanel), and the nodal lines corresponded to the lines of functional conduction block as shown in Figures 5(d) and 5(e). In the HF group, VF was converted to ventricular tachycardia (VT) in 2 hearts and converted to sinus rhythm in 1 heart.

**Figure 6:**
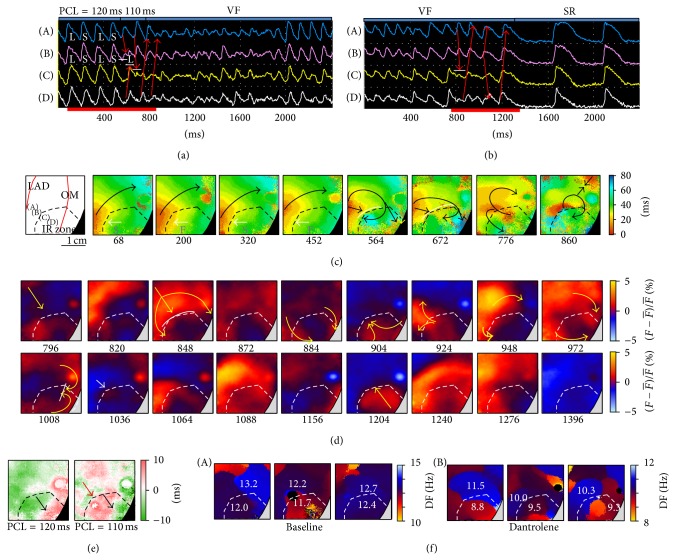
VF induction and spontaneous termination during dantrolene administration in a non-HF heart. (a) and (b) *V*_*m*_ tracings corresponding to the points labeled in panel (c) during VF induction and VF termination, respectively. The red arrows indicate wavefront propagation, and the white lines indicate conduction block. L: long; S: short. (c) Isochronal maps corresponding to the period labeled in panel (a) showing CV alternans followed by conduction block and VF initiation. The white lines indicate functional conduction block. (d) Frame shots corresponding to the period labeled in panel. (b) The white lines indicate lines of functional conduction block, and the white arrow indicates focal breakthrough. (e) APD difference maps showing SDA induction. The black and red arrows indicate nodal lines. (f) The dominant frequency (DF) maps of induced VF at baseline (A) and after dantrolene infusion (B). SR: sinus rhythm.

In the non-HF group, VF was converted to VT and then stopped (*n* = 4) or directly converted to sinus rhythm (*n* = 2) in 6 hearts with nonsustained VF after dantrolene infusion.  Figure 6 shows an example. At baseline, sustained VF was induced as PCL decreased to 100 ms, and electrical defibrillation was needed to convert it (data not shown). After dantrolene infusion, VF was induced at a longer PCL but terminated spontaneously 49 seconds after induction. As shown in Figure 6(a) (*V*_*m*_ tracings), APD alternans was induced (at PCL = 120 ms) before VF induction. The corresponding isochronal maps (Figure 6(c)) show pacing-induced CV alternans and crowded isochronal lines (white arrows, frames 68 to 452 ms) at PCL = 120 ms. A longer APD was correlated to a slower CV, indicating that CV was controlled at least partly by APD (ERP) at faster pacing rates. When PCL was shortened to 110 ms, the pacing wavefront met a line of functional conduction block (frames 564 and 672 ms) to form reentry and perpetuated VF (frames 776 and 860 ms) (online video 3).  Figure 6(b) (*V*_*m*_ tracings) and Figure 6(d) (snapshots) show spontaneous VF termination. A wavefront met a line of functional conduction block to form figure-8 reentry (snapshots 796 to 948 ms), but the new-formed wavefronts were blocked (snapshot 1008 ms), followed by a wavefront arising from the border of the IR zone (white arrow, snapshot 1036 ms) and then a wavefront from the border of the mapping field (snapshots 1204 to 1396 ms) before conversion to sinus rhythm (online video 4).  Figure 6(e) shows that SDA was induced by rapid pacing (PCL 120 ms, left subpanel), and a new nodal line (red arrow, right subpanel) was formed as the PCL decreased to 110 ms. These nodal lines corresponded to the lines of functional conduction block shown in Figures 6(c) and 6(d) and played an important role in wavelet generation and annihilation.

The DF_max⁡_ of VF was significantly decreased by dantrolene infusion (Table 1). In the HF group, the difference of DF_max⁡_ between the IR and non-IR zones was not significant at baseline (*P* = 0.23, *n* = 9, Table 2); however, it became significant with dantrolene infusion (*P* = 0.008, *n* = 9, Table 2). In the non-HF group, the IR zone had a lower DF_max⁡_ than the non-IR zone at baseline (*P* = 0.01, *n* = 9) and after dantrolene infusion (*P* = 0.009, *n* = 9, Table 2). As shown in Figure 6(f), the mean values of the DF_max⁡_ of VF were 12.4 ± 0.5 Hz at baseline and 9.9 ± 0.9 Hz with dantrolene. The non-IR zone had a higher mean DF_max⁡_ of VF than that in the IR zone at baseline (12.7 ± 0.5 Hz versus 12.0 ± 0.4 Hz) and with dantrolene infusion (10.6 ± 0.8 Hz versus 9.2 ± 0.4 Hz). This suggests a driven rather than a driver role of the IR zone in VF dynamics in this model.

## 4. Discussion

We studied the effects of dantrolene on suppression of VPB and VF inducibility in post-IR injury hearts with and without pacing-induced HF. The major results were that dantrolene did not suppress spontaneous VPBs and VF inducibility in either group. Dantrolene exerted antiarrhythmic action via prolonging APD and ERP, which may underlie a higher VF conversion rate in smaller hearts in the non-HF group. But the APD and ERP lengthening effect also intensified the rate-dependent conduction delay; in addition, dantrolene steepened APD restitution properties and facilitated both spatially concordant and discordant alternans induction, all of which may oppose its antiarrhythmic effects.

### 4.1. VPB Suppression in IR Hearts

We previously reported that dantrolene was effective in suppressing VPBs via decreasing diastolic SR Ca^2+^ oscillations in an AMI with HF model [9]. In post-IR injury hearts, Sharma et al. reported that reperfusion for 30 minutes resulted in a significant increase in tissue Ca^2+^ in the IR zone [18]. Since a reduced binding affinity of calmodulin to ryanodine receptor-2 fitted for dantrolene's action on SR is found in failing hearts rather than normal hearts [19], dantrolene would be predicted to suppress VPBs in the IR model, especially in the HF group. However, our data did not show a decreased VPB burden during dantrolene infusion neither in HF group nor in non-HF group. There are some possible explanations. First, as Vera et al. reported [20], triggered activity may not be the dominant mechanism responsible for VPBs in IR hearts. Yee et al. noted only small-amplitude oscillatory afterpotentials during reperfusion but no triggered activity, which cast doubt on whether triggered activity is a mechanism of reperfusion arrhythmias [21]. On the other hand, enhanced automaticity has been reported to be the dominant mechanism of VPBs in post-IR injury hearts [22]. The lack of a VPB suppressing effect by dantrolene could be due to its inability to suppress automaticity [23, 24]. Second, if triggered activity is the dominant mechanism of VPBs, it implies that dantrolene could not ameliorate the Ca_i_ overload condition effectively in these IR hearts. Temsah et al. reported that the attenuated recovery of contractile function and Ca_i_ overload post-IR injury are due to reduced SR Ca^2+^ uptake, Ca^2+^ release, and ryanodine-binding activities [25]. Therefore, suppression of diastolic SR Ca^2+^ leakage alone may not be sufficient to improve Ca_i_ overload conditions.

### 4.2. Dantrolene on Conduction Delay in the IR Rabbit Model

Penkoske et al. reported that reperfusion arrhythmias are initiated by enhanced pacemaker activity and maintained by localized and slowed conduction within IR regions [26]. Both the IR-induced downregulation of Na^+^ channel [27] and reduced gap junction conductance as a result of the elevated Ca_i_ of the IR myocardium may account for a depressed conduction in the IR region [28]. Salata et al. reported that dantrolene has no significant effect on upstroke velocity of phase 0 but prolongs APD mediated in part by a decrease in the intracellular free Ca^2+^ concentration [23, 29]. Therefore, dantrolene can either ameliorate conduction depression by decreasing intracellular free Ca^2+^ concentration (to improve gap junction conductance) or further decelerate CV via prolonging APD (as impulses traveling through partially refractory tissue). Our data showed that dantrolene deteriorated conduction delay and led to frequency-dependent functional conduction block, which may play a role in facilitating VF induction (as shown in Figures 5 and 6). However, the worsening of conduction delay was caused by APD prolongation, which neutralized its arrhythmogenicity by hindering wavelet propagation to stop VF (as shown in Figure 6).

### 4.3. Anti- and Proarrhythmic Effects of Dantrolene on IR Hearts

Dantrolene prolongs APD, which by itself can decrease the DF_max⁡_ of VF and influence the persistence of VF through its effects on wavelength [30]. However, the electrophysiological studies and mapping data also showed that dantrolene has proarrhythmic effects (i.e., slowing CV, steepening APD restitution slope, and lowering the pacing threshold of arrhythmogenic alternans induction) in the IR model. These effects have been demonstrated to create functional electrophysiological dispersion that destabilizes wave propagation and enhances the tissue vulnerability to initiate reentry [31, 32]. The lack of significant effects of dantrolene on the VF inducibility and maintenance in the HF group might be due to a counterbalance of its antiarrhythmic and proarrhythmic effects. On the other hand, dantrolene had a higher VF conversion rate in the non-HF group, which is likely due to a greater APD prolonging effect in smaller hearts in the non-HF group compared with the HF group. Salata et al. demonstrated that dantrolene prolongs APD, possibly mediated by inhibiting L-type Ca^2+^ channels, subsequently depressing Ca^2+^-sensitive potassium channels [29]. Because L-type Ca^2+^ channels are impaired in pacing-induced HF [33], it is possible that fewer L-type Ca^2+^ channels are available to respond to the inhibitory effect of dantrolene, resulting in a less degree of APD prolongation in the HF group.

### 4.4. Clinical Implications

Dantrolene has been shown to prevent VF storm in failing hearts with AMI [9]. However, the presented data suggest that dantrolene has no significant effects on VF inducibility and maintenance in failing hearts post-IR injury. The clinical benefits of continuous dantrolene use in HF patients with AMI and VF storm postreperfusion therapy are not obvious and may require further investigations.

### 4.5. Study Limitations

Given that the electrophysiological studies were performed with Langendorff perfusion, it is possible that electrophysiological parameters may be different when the hearts are in situ and also caution is advised when extrapolating our model to clinical IR cardiac injury. Because the Ca^2+^ amplitude of optical signals was a ratio derived from the difference of Ca^2+^ dye fluorescence intensity between the peak and the trough of acquired beats rather than the absolute value of intracellular Ca^2+^ concentration changes, we could not make comparisons of the absolute Ca^2+^ amplitude between the optical signals acquired at different moments (baseline versus after dantrolene). Therefore, whether the amplitude of Ca^2+^ transient was affected by dantrolene was not shown in this study. The estimated CV slowing in the IR zone might be underestimated since the initial portion of IR distance measurement (from the earliest activation point to the IR zone border) was in the non-IR zone. We did not perform patch clamp studies and measure gap junction conductance; therefore, whether dantrolene exerted a direct inhibition effect on sodium current and/or gap junction coupling was not shown in this study.

## Supplementary Material

Online video 1: VF induction by rapid pacing during dantrolene infusion in a failing heart.
Online video 2: VF converted to VT during dantrolene infusion in a failing heart.
Online video 3: VF induction by rapid pacing during dantrolene infusion in a non-failing heart.
Online video 4: VF converted to sinus rhythm during dantrolene infusion in a non-failing heart.

## Figures and Tables

**Table 1:** Electrophysiological effects of dantrolene in isolated Langendorff-perfused hearts after IR injury in the HF and non-HF groups.

	CV_(300)_ (cm/s)	CV_(200)_ (cm/s)	CV_(150)_ (cm/s)	CV_(120)_ (cm/s)	APD_(300)_ (ms)	APD_(200)_ (ms)	APD_(150)_ (ms)	ERP (ms)	DF_max⁡_ (Hz)	APDR slope	Ca_i_ decay (*τ*) (ms)
HF (*n* = 9)											
Baseline	72 ± 12	68 ± 11	63 ± 11	55 ± 9	157 ± 16	130 ± 11	108 ± 10	161 ± 27	14.2 ± 1.3	1.14 ± 0.17	69 ± 12
Dantrolene	67 ± 10^*^	61 ± 11^*^	55 ± 10^*^	47 ± 6^*^	169 ± 20^*^	135 ± 12^*^	110 ± 8^*^	182 ± 12^*^	11.3 ± 1.5^*^	1.34 ± 0.24^*^	72 ± 10
Non-HF (*n* = 9)											
Baseline	81 ± 7	76 ± 9	70 ± 9	64 ± 8	148 ± 9	125 ± 9	107 ± 12	142 ± 11	13.0 ± 1.7	0.97 ± 0.19	66 ± 8
Dantrolene	73 ± 11^*^	67 ± 11^*^	61 ± 12^*^	55 ± 11^*^	160 ± 14^*^	134 ± 11^*^	112 ± 12^*^	170 ± 10^*^	10.4 ± 1.5^*^	1.19 ± 0.30^*^	67 ± 8

Values are mean ± SD. APD_(300)_, APD_(200)_, and APD_(150)_, action potential duration at pacing cycle lengths of 300, 200, and 150 ms; CV_(300)_, CV_(200)_, CV_(150)_, and CV_(120)_, conduction velocity at pacing cycle lengths of 300, 200, 150, and 120 ms; DF_max⁡_: the maximal dominant frequency of ventricular fibrillation; Ca_i_: intracellular Ca^2+^; ERP: effective refractory period; APDR slope: the mean maximum slope of APD restitution curves.

Asterisks denote values significantly different from the baseline.

**Table 2:** Electrophysiological effects of dantrolene in non-IR and IR zones in HF and non-HF groups.

	CV_(300)_ (cm/s)	CV_(200)_ (cm/s)	CV_(150)_ (cm/s)	CV_(120)_ (cm/s)	APD_(300)_ (ms)	APD_(200)_ (ms)	APD_(150)_ (ms)	DF_max⁡_ (Hz)	Ca_i_ decay (*τ*) (ms)	APDR slope
HF (n = 9)										
Baseline										
Non-IR	76 ± 11	72 ± 12	66 ± 11	57 ± 7	155 ± 13	130 ± 11	109 ± 10	14.4 ± 1.6	67 ± 11	1.14 ± 0.16
IR	70 ± 9	65 ± 9	60 ± 9	55 ± 9	158 ± 18	130 ± 11	108 ± 11	14.0 ± 1.3	70 ± 14	1.13 ± 0.20
Dantrolene										
Non-IR	73 ± 9	66 ± 10	61 ± 9	51 ± 6	167 ± 19	135 ± 11	110 ± 6	11.7 ± 1.2	72 ± 10	1.34 ± 0.25
IR	67 ± 8	61 ± 10	54 ± 9	45 ± 6^*^	168 ± 22	134 ± 12	109 ± 8	10.9 ± 1.8^*^	73 ± 10	1.35 ± 0.26
Non-HF (n = 9)										
Baseline										
Non-IR	84 ± 6	79 ± 7	73 ± 5	69 ± 5	148 ± 9	125 ± 9	107 ± 11	13.7 ± 1.3	63 ± 6	1.02 ± 0.16
IR	78 ± 7	73 ± 9	67 ± 7	63 ± 7	149 ± 9	125 ± 9	108 ± 12	12.3 ± 2.2^*^	69 ± 8^*^	0.91 ± 0.20
Dantrolene										
Non-IR	78 ± 8	70 ± 8	66 ± 7	60 ± 8	162 ± 15	134 ± 11	113 ± 12	11.1 ± 1.5	65 ± 7	1.29 ± 0.38
IR	67 ± 11^*^	60 ± 9^*^	52 ± 10^*^	48 ± 9^*^	162 ± 15	134 ± 11	111 ± 11	9.7 ± 1.7^*^	70 ± 9^*^	1.09 ± 0.25

Values are mean ± SD. APD_(300)_, APD_(200)_, and APD_(150)_, action potential duration at pacing cycle lengths of 300, 200, and 150** **ms; CV_(300)_, CV_(200)_, CV_(150)_, and CV_(120)_, conduction velocity at pacing cycle lengths of 300, 200, 150, and 120** **ms; DF_max⁡_: the maximal dominant frequency of ventricular fibrillation; Ca_i_: intracellular Ca^2+^; CV: conduction velocity.

Asterisks denote values significantly different between non-IR and IR zones.
